# Identification of immune-related genes in diagnosing retinopathy of prematurity with sepsis through bioinformatics analysis and machine learning

**DOI:** 10.3389/fgene.2023.1264873

**Published:** 2023-11-10

**Authors:** Han Chen, Enguang Chen, Yao Lu, Yu Xu

**Affiliations:** ^1^ Department of Ophthalmology, School of Medicine, Xinhua Hospital, Shanghai Jiao Tong University, Shanghai, China; ^2^ Department of Ophthalmology, Anhui No. 2 Provincial People’s Hospital, Anhui, Hefei, China

**Keywords:** retinopathy of prematurity, sepsis, machine learning, immune infiltration, diagnostic value

## Abstract

**Background:** There is increasing evidence indicating that immune system dysregulation plays a pivotal role in the pathogenesis of retinopathy of prematurity (ROP) and sepsis. This study aims to identify key diagnostic candidate genes in ROP with sepsis.

**Methods:** We obtained publicly available data on ROP and sepsis from the gene expression omnibus database. Differential analysis and weighted gene correlation network analysis (WGCNA) were performed to identify differentially expressed genes (DEGs) and key module genes. Subsequently, we conducted functional enrichment analysis to gain insights into the biological functions and pathways. To identify immune-related pathogenic genes and potential mechanisms, we employed several machine learning algorithms, including Support Vector Machine Recursive Feature Elimination (SVM-RFE), Least Absolute Shrinkage and Selection Operator (LASSO), and Random Forest (RF). We evaluated the diagnostic performance using nomogram and Receiver Operating Characteristic (ROC) curves. Furthermore, we used CIBERSORT to investigate immune cell dysregulation in sepsis and performed cMAP analysis to identify potential therapeutic drugs.

**Results:** The sepsis dataset comprised 352 DEGs, while the ROP dataset had 307 DEGs and 420 module genes. The intersection between DEGs for sepsis and module genes for ROP consisted of 34 genes, primarily enriched in immune-related pathways. After conducting PPI network analysis and employing machine learning algorithms, we pinpointed five candidate hub genes. Subsequent evaluation using nomograms and ROC curves underscored their robust diagnostic potential. Immune cell infiltration analysis revealed immune cell dysregulation. Finally, through cMAP analysis, we identified some small molecule compounds that have the potential for sepsis treatment.

**Conclusion:** Five immune-associated candidate hub genes (CLEC5A, KLRB1, LCN2, MCEMP1, and MMP9) were recognized, and the nomogram for the diagnosis of ROP with sepsis was developed.

## 1 Introduction

Sepsis is a severe systemic infection characterized by a dysregulated host response, leading to organ dysfunction to varying degrees (Singer et al., 2016). Furthermore, according to population-level studies over the past 2 decades, the occurrence of neonatal sepsis was recorded at 2.2%, with mortality rates spanning from 11% to 19% (Liu et al., 2016; Dong et al., 2020). In summary, sepsis stands as one of the primary contributors to hospital-related mortality (Rhee et al., 2019). Additionally, it imposes significant economic burden and psychological stress on patients and their families.

Retinopathy of prematurity (ROP) constituted a significant factor that led to visual impairment and blindness in children, characterized by abnormal retinal neovascularization in premature infants (Rivera et al., 2011). It was recognized as an “iatrogenic disease” following the introduction of supplemental oxygen and incubators, which made survival of premature infants possible (Dogra et al., 2017). The incidence and severity of ROP were impacted by multiple factors, such as birth weight, gestational age, oxygen exposure, bronchopulmonary dysplasia, blood transfusions, as well as several systemic risk factors (Wu et al., 2018).

Inflammation is widely recognized as a significant factor in the development of ROP. Furthermore, in the oxygen-induced retinopathy model, it has been demonstrated that systemic inflammation in newborns disrupts the development of retinal blood vessels and induces the pathological features of ROP (Tremblay et al., 2013; Hong et al., 2014). Premature infants are at increased risk of developing ROP due to incomplete development, especially in the vascular system of the eyes (Sato et al., 2009). Sepsis, as a systemic infection, can lead to vascular inflammation and endothelial dysfunction, thereby increasing the risk of ROP in premature infants (Lee and Dammann, 2012). Premature infants with low birth weight were more susceptible to infections, and there was evidence of an increased risk of ROP in these cases. Several studies have demonstrated an association between the incidence of early-onset sepsis in premature infants and an elevated risk as well as severity of ROP (Huang et al., 2019; Bonafiglia et al., 2022; Shen et al., 2022). This indicates that infections and inflammatory responses may have a vital role in the pathogenesis of ROP. Currently, the mechanisms underlying the role of inflammation in ROP accompanied by sepsis are still under investigation.

In recent years, bioinformatics analysis has found widespread applications in exploring potential pathogenic mechanisms, identify therapeutic targets, and discover small molecule drugs for various diseases. In our study, we utilized sepsis and ROP datasets downloaded from GEO to identify differentially expressed genes (DEGs) and important module genes through weighted gene co-expression network analysis (WGCNA). We conducted enrichment analysis on intersecting genes, constructed PPI networks, and applied machine learning algorithms, including LASSO, RF, and SVM-RFE. We performed immune cell infiltration analysis and Connectivity map (cMAP) analysis. Furthermore, we assessed the essential immune-related diagnostic genes for sepsis combined with ROP using nomogram and ROC curve analysis. This research aims to identify potential immune-related diagnostic biomarkers for ROP with sepsis, providing valuable insights for clinical diagnosis and treatment guidance.

## 2 Materials and methods

### 2.1 Data collection

**FIGURE 1:**
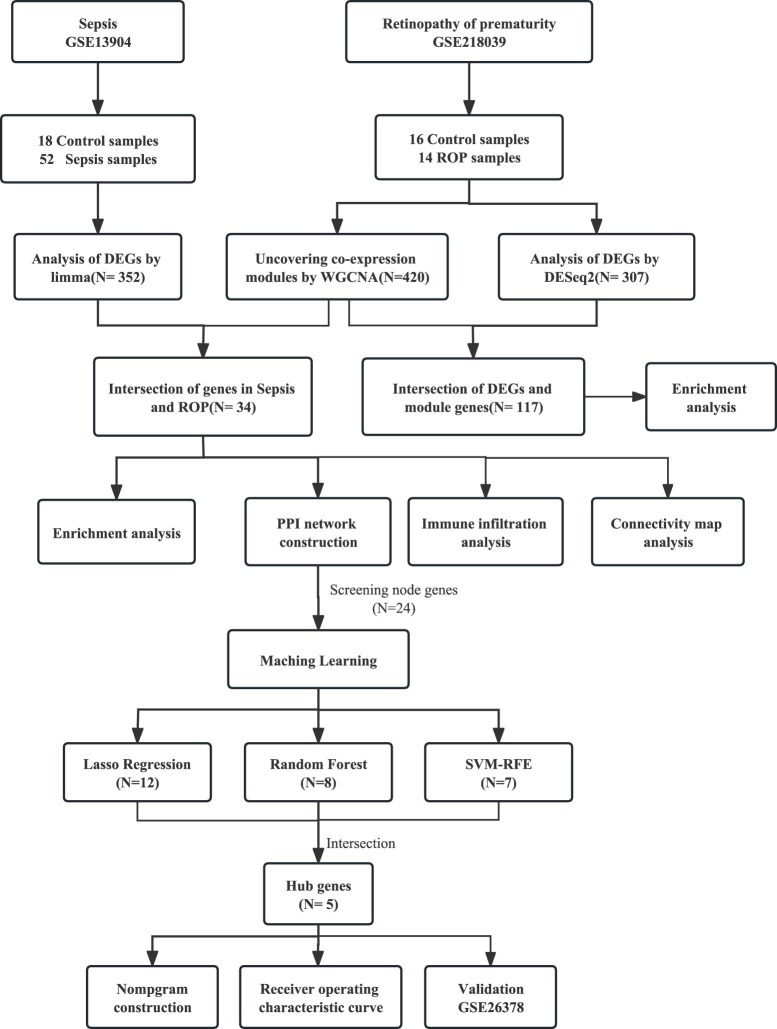
Study flowchart.

We performed a search using “retinopathy of prematurity” and “sepsis” as keywords in the GEO database and retrieved two relevant gene expression datasets: GSE13904 and GSE218039 (Barrett et al., 2013). The microarray dataset GSE13904, comprises 18 samples from normal children and 52 samples from sepsis cases. The RNA-seq-based dataset GSE218039, consists of 16 control samples and 14 samples from ROP cases. To better illustrate our research process, we have created a flowchart in the Figure 1.

### 2.2 Data preprocessing and differentially expressed genes analysis

The microarray dataset for GSE13904 was downloaded from GEO and subjected to normalization using the “limma” package in R software. The “limma” package was employed to compare the gene expression profiles between normal samples and sepsis cases in order to identify DEGs. For GSE218039, we obtained the raw expression data, normalized the data, and employed the DESeq2 package in R to identify DEGs. Probe sets without corresponding gene symbols were excluded. Genes with multiple probe sets were averaged. Genes with an adjusted *p*-value <0.05 and a threshold of |log2 Fold Change| > 1 were set for the analysis. Furthermore, we employed the R software package “ggplot2” to create separate volcano plots for visualizing the DEGs obtained from each of the two datasets.

### 2.3 Weighted gene correlation network analysis (WGCNA)

To investigate the correlation between genes, we constructed a gene co-expression network using the WGCNA (Langfelder and Horvath, 2008). Firstly, we calculated the median absolute deviation (MAD) for each gene and identified the top 5,000 genes. Then, we computed the adjacency using a “soft” thresholding power (*β*) derived from co-expression similarity. The adjacency was transformed into a topological overlap matrix (TOM), and gene dissimilarity and heterogeneity were assessed. Thirdly, module detection was performed using hierarchical clustering and dynamic tree cutting. Genes that displayed comparable expression profiles were assigned to gene modules utilizing the average linkage hierarchical clustering method, ensuring a minimum gene module size (*n* = 30) based on the TOM-based dissimilarity measure and gene dendrogram. Finally, employing WGCNA analysis, we identified and visualized the crucial modules associated with ROP.

### 2.4 Function enrichment analysis

To explore the functional and interactive roles of genes in biological pathways, we utilized the DAVID online database (https://david.ncifcrf.gov) to perform GO and KEGG pathway enrichment analyses (Kanehisa and Goto, 2000; The Gene Ontology Consortium, 2019). In this study, the results were visualized using the bioinformatics platform (https://www.bioinformatics.com.cn). We performed two rounds of GO and KEGG analysis based on the intersection of DEGs and the key module genes of ROP, and the intersection of sepsis-related DEGs and the key module genes of ROP.

### 2.5 Protein–protein intersection network construction

We uploaded the candidate genes to the STRING database (www.string-db.org) and constructed a PPI network (Szklarczyk et al., 2021), with a medium confidence score of >0.4. Subsequently, we utilized the Cytoscape software to identify interacting genes for further analysis (Shannon et al., 2003).

### 2.6 Machine learning

To further identify candidate biomarkers for ROP and sepsis and establish a diagnostic model, we employed three machine learning algorithms. Lasso is a regularization method for linear regression that incorporates feature selection and model interpretability by adding an L1 regularization term, thereby improving prediction accuracy (Yang et al., 2018). RF is an ensemble learning algorithm that constructs multiple decision trees for classification or regression (Ellis et al., 2014). The final prediction result is based on the voting or average of all decision trees, providing robustness and generalization capabilities. SVM-RFE combines support vector machines with recursive feature elimination, eliminating features with minimal impact on the classification results to obtain a simpler and superior performing model (Sanz et al., 2018). The genes identified by the overlapping of these three machine learning algorithms serve as candidate hub genes for diagnosis.

### 2.7 Nomogram construction and validation of candidate biomarkers

A nomogram for candidate hub genes was constructed using the “rms” package in R. Additionally, we utilized ROC analysis to further assess the clinical value of the candidate hub genes and the nomogram. The clinical value was determined by the area under the curve (AUC). An AUC value greater than 0.7 was deemed to indicate excellent diagnostic value.

To validate the robustness of the hub genes analysis results, we conducted validation using the microarray dataset GSE26378, which comprises 21 control samples and 82 sepsis cases. Statistical significance was determined by an unpaired *t*-test with a *p*-value <0.05.

### 2.8 Immune infiltration analysis

The relative abundance of immune-infiltrating cells was calculated using the CIBERSORT algorithm from the “IOBR” R package (Newman et al., 2015; Zeng et al., 2021). A bar plot was generated to illustrate the proportions of immune cells in different samples, and a box plot was employed to compare the different types of immune cells between the sepsis and control groups. Additionally, the “ggplot” R package was used to visualize the correlations between the hub genes and the 22 infiltrating immune cells.

### 2.9 Connectivity map (cMAP) analysis

The cMAP (https://clue.io) is an important gene expression database that provides valuable resources for researchers to explore the associations between genes, drugs, diseases, and small molecules (Subramanian et al., 2017). In this study, the overlapping upregulated genes from the DEGs of sepsis and the key modules of ROP were included in the cMAP online database to identify potential small molecule drugs that could be used for treatment. Finally, the top 10 small molecule compounds with the most negative correlation were determined.

### 2.10 Statistical analysis

All statistical analyses were performed using R software. The Wilcoxon or Student’s t-test was utilized to analyze the difference between the two groups. The correlation analysis between the variables was determined using the Spearman’s correlation test. Statistical significance was set at a two-tailed *p* < 0.05.

## 3 Results

### 3.1 Differentially expressed genes

**FIGURE 2:**
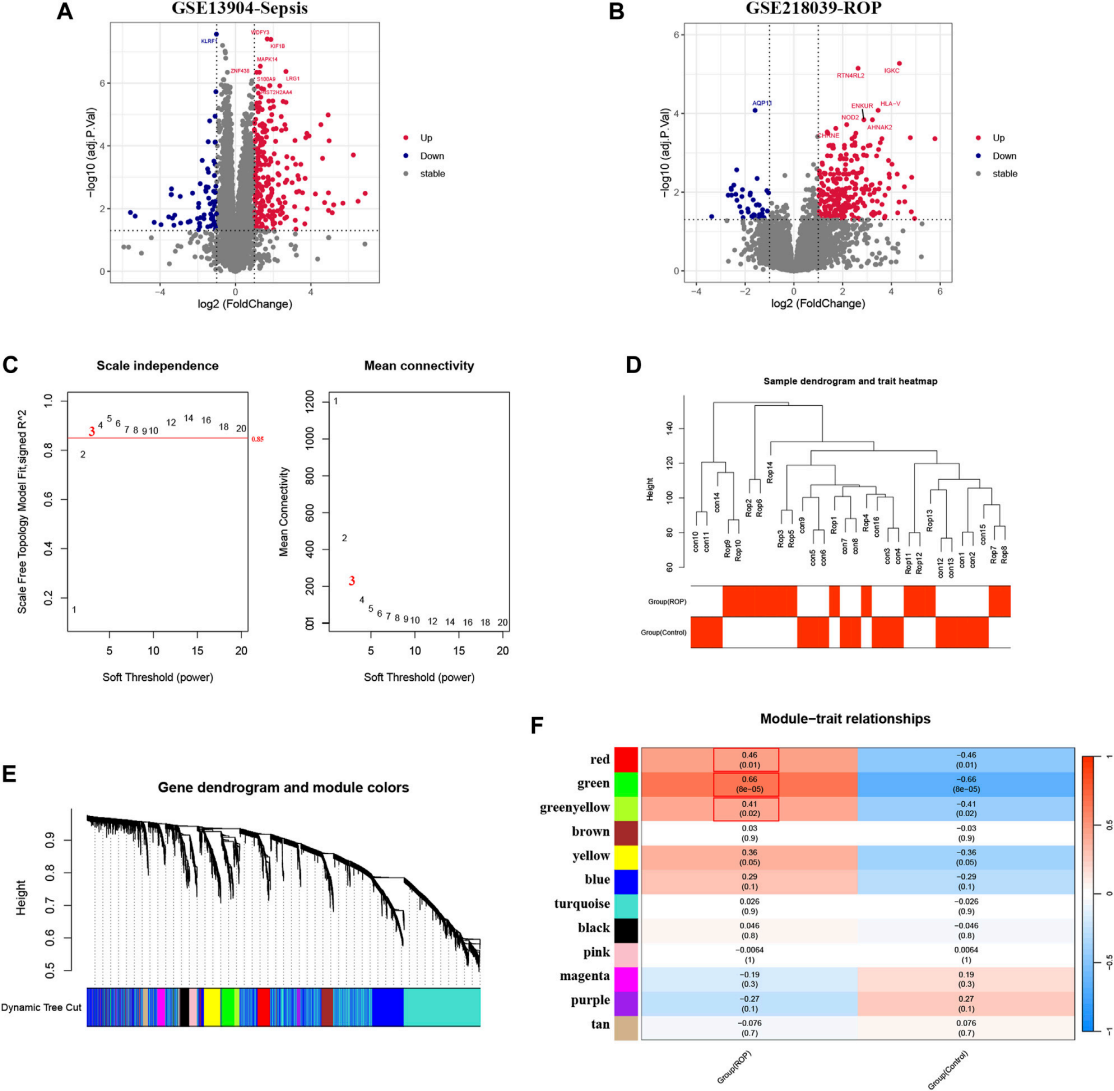
Analysis of DEGs in Sepsis and ROP datasets, and identification of critical modules using WGCNA in ROP. **(A)** The volcano plot revealing DEGs in the Sepsis dataset. **(B)** The volcano plot revealing DEGs in the ROP dataset. **(C)** *β* = 3 is selected as the soft threshold with the combined analysis of scale independence and average connectivity. **(D)** Sample dendrogram and trait heatmap. **(E)** Gene co-expression modules represented by different colors under the gene tree. **(F)** The heatmap revealing the relationship between modules and status of ROP. The correlation (upper) and *p*-value (bottom) of module eigengenes and status of ROP were presented.

Overall, 352 DEGs were identified in the sepsis dataset, with 266 genes significantly upregulated and 86 genes significantly downregulated. As for the ROP dataset, a total of 307 DEGs were identified, with 267 genes upregulated and 40 genes downregulated. The volcano plots of DEGs are shown in Figures 2A, B.

### 3.2 Weighted gene co-expression network analysis and selection of critical modules

In GSE218039, we performed WGCNA to identify significantly correlated gene modules in ROP. In order to determine the most suitable soft threshold ensuring a scale-free network, we utilized the “pick Soft Threshold” function from the WGCNA package, which filtered power parameters within the range of 1–20. Based on a scale independence value greater than 0.85, we selected a power value (β) of 3, which corresponded to a scale-free *R* ^2^ of 0.88, as the optimal soft threshold (Figure 2C). Figure 2D illustrates the dendrogram of clustering for ROP and control groups. We used the “cutree” dynamic and module eigengenes functions to create a cluster diagram. This analysis resulted in the identification of 12 modules, each composed of genes exhibiting similar co-expression patterns (Figure 2E). Then, a heatmap was generated to display the correlation between ROP and modules based on Spearman correlation coefficients (Figure 2F). Notably, three modules—“red”, “green”, and “greenyellow”—displayed robust positive correlations with ROP and were therefore designated as ROP-related modules, with respective correlation coefficients and *p*-values as follows: red module (*r* = 0.46, *p* = 0.01), green module (*r* = 0.66, p = 8e-05), and greenyellow module (*r* = 0.41, *p* = 0.02). These modules consist of 167, 186, and 67 genes, totaling 420 genes in all, collectively considered as key module genes associated with ROP.

### 3.3 Functional enrichment analysis of ROP

**FIGURE 3:**
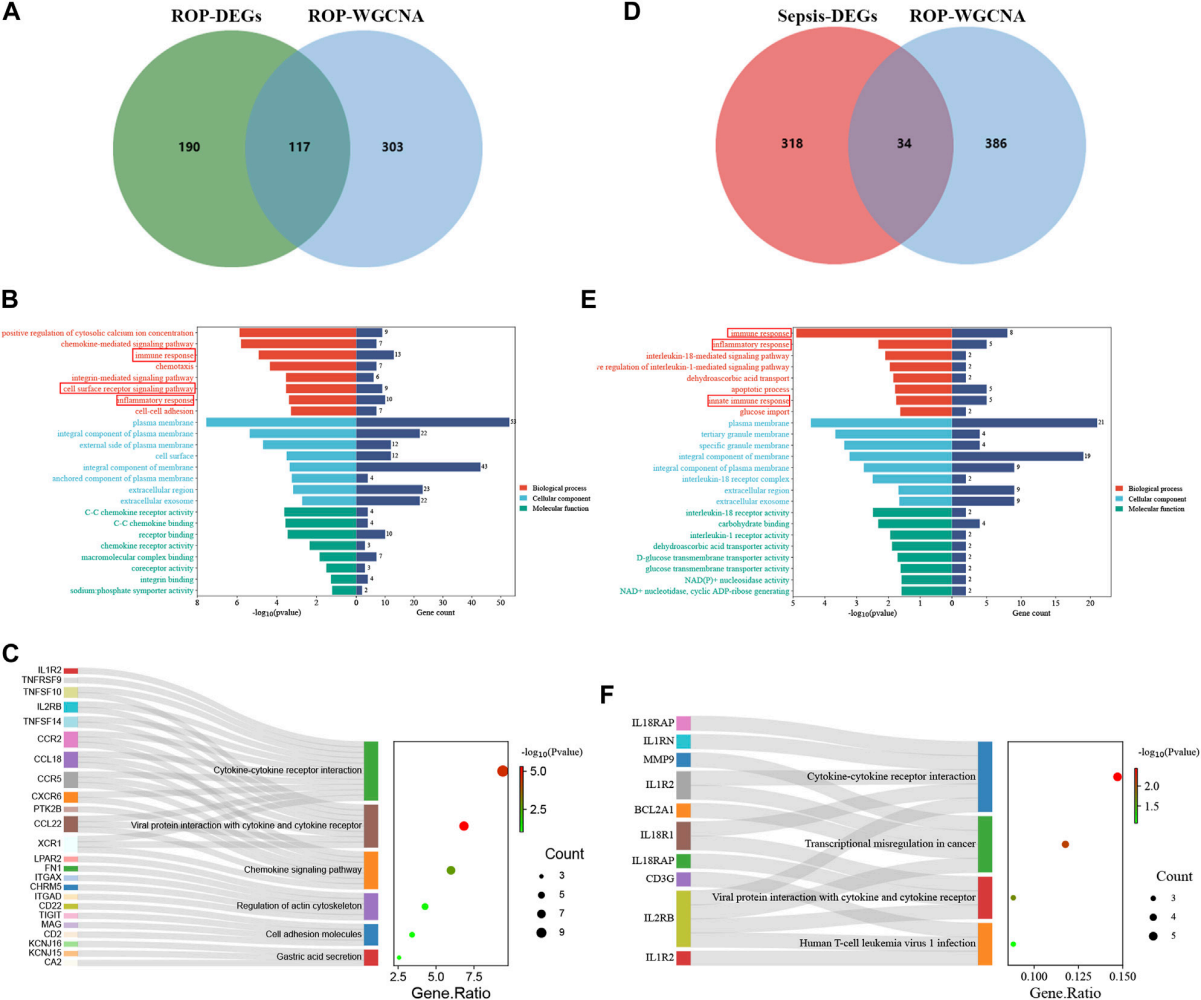
Enrichment analysis. **(A)** A total of 117 genes in ROP were identified from the intersection of DEGs and crucial module genes via the venn diagram **(B)** The GO analysis of the intersection of genes in ROP. **(C)** KEGG pathway analysis of the intersection of genes in ROP. **(D)** A total of 34 genes are identified from the intersection of sepsis-related DEGs with ROP key module genes via the venn diagram. **(E)** GO analysis of 34 common genes. **(F)** KEGG analysis of 34 common genes.

Given the rare nature of ROP patients, we conducted an in-depth analysis of the dataset GSE218039 to explore potential mechanisms underlying ROP. Furthermore, this dataset is relatively new and has not been previously explored. We further conducted functional enrichment analysis based on the intersection of differential analysis results and key module genes from WGCNA. By intersecting the 307 DEGs and the 420 in the key module genes, a total of 117 common genes were screened (Figure 3A). As depicted in Figure 3B, Biological Process (BP) terms were predominantly enriched in “immune response”, “cell surface receptor signaling pathway”, and “inflammatory response”. In terms of Cellular Component (CC) ontology, enrichment was observed in “plasma membrane”, and “external side of plasma membrane”. Regarding Molecular Function (MF) analysis, enrichment was mainly found in “receptor binding”, and “C-C chemokine receptor activity”. In addition, as shown in Figure 3C, KEGG analysis revealed enrichment in “Viral protein interaction with cytokine and cytokine receptor” and “Cytokine-cytokine receptor interaction”.

The enrichment analysis reveals a significant correlation between the overlapping genes in ROP and immune response and inflammation, suggesting their potential involvement in the pathogenesis of ROP. The detailed results of the GO and KEGG analyses from the DAVID database are available in Supplementary Table S1.

### 3.4 Enrichment analysis of ROP with sepsis and screening node genes via the PPI network

To explore the potential relationship between ROP-associated crucial genes and the pathogenesis of sepsis, we identified 34 genes from the intersection of sepsis-related DEGs with ROP key module genes using a Venn diagram (Figure 3D). GO analysis highlighted that these genes were primarily enriched in “immune response”, “inflammatory response”, and “innate immune response” (BP); “plasma membrane” and “interleukin-18 receptor complex” (CC); “interleukin-18 receptor activity” and “interleukin-1 receptor activity” (MF) (Figure 3E). Furthermore, based on KEGG analysis, the 34 genes showed prominent enrichment in pathways such as “Cytokine-cytokine receptor interaction” and “Viral protein interaction with cytokine and cytokine receptor” (Figure 3F, Supplementary Table S2). All the above findings strongly implicate the involvement of the immune system in these processes.

**FIGURE 4:**
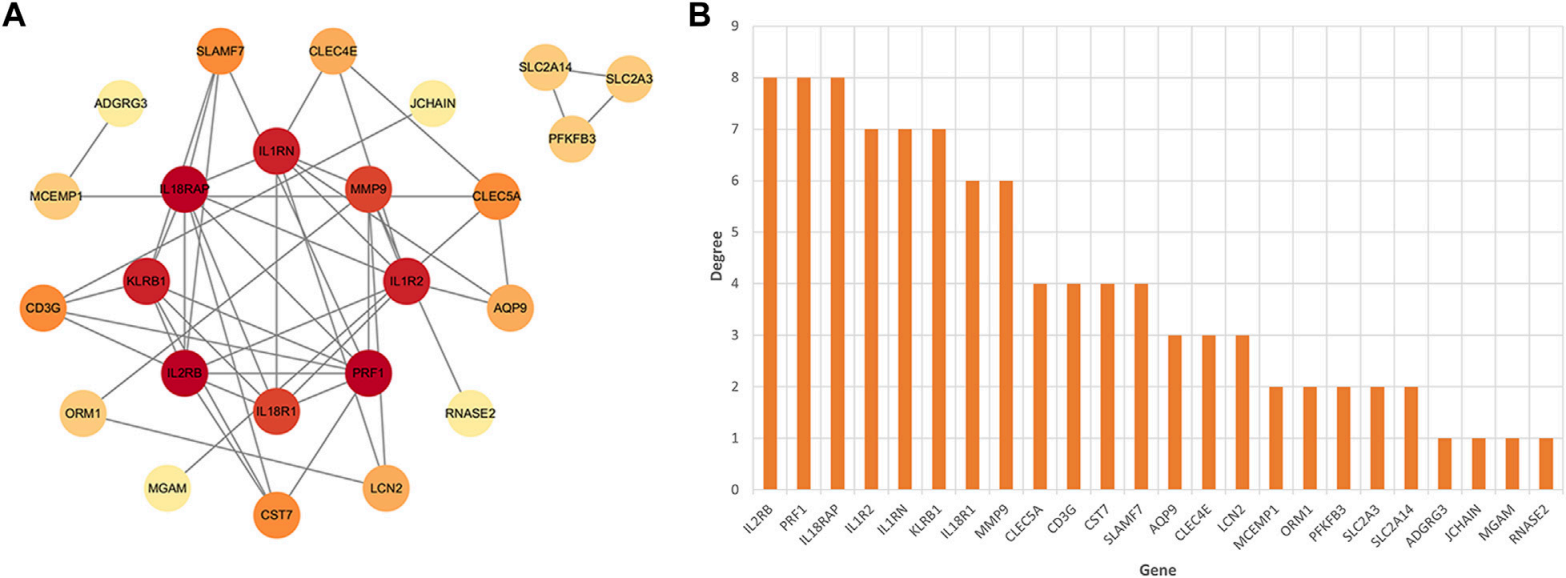
The identification of node genes from PPI network. **(A)** The PPI network demonstrates that 24 genes interact with each other. **(B)** The column shows the gene nodes of 24 genes in the PPI network.

After identifying the immune-related nature of these genes, we constructed a PPI network to explore the interacting nodes among them (Figure 4A). Using Cytoscape software (Figure 4B), we identified 24 genes that interacted with each other, and these genes were sorted by their node degree.

### 3.5 Screening key genes via machine learning

**FIGURE 5:**
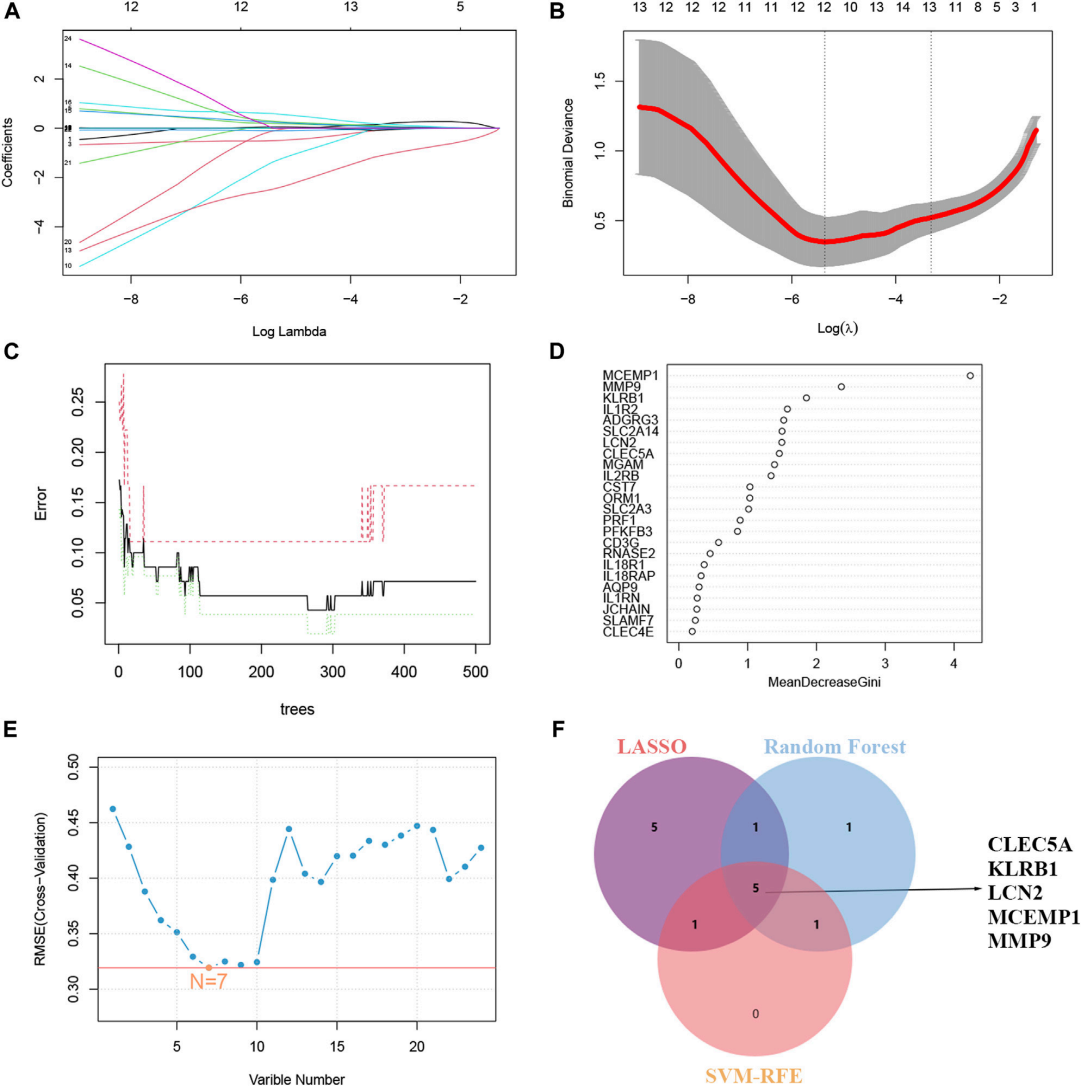
Machine learning in screening candidate hub genes for ROP with sepsis. **(A, B)** The number of genes (*n* = 12) corresponding to the lowest point of the curve in the LASSO model. **(C)** When “tree” was set to 200, the error within the model had essentially stabilized. **(D)**The Random Forest algorithm ranked genes based on the MDG. **(E)** Based on SVM-RFE to screen biomarkers. **(F)** Venn plot exhibiting the reliable biomarkers among LASSO, RF, and SVM-RFE.

Potential candidate hub genes were screened using the LASSO regression, RF algorithm, and SVM-RFE. Notably, LASSO regression revealed 12 potential candidate hub genes (Figures 5A, B). The RF algorithm ranked candidate genes based on the Mean Decrease Gini and selected the top eight genes with the highest scores (Figures 5C, D). SVM-RFE identified 7 genes as candidate biomarkers (Figure 5E). By employing a Venn diagram (Figure 5F), we intersected the results from the three algorithms, ultimately identifying five hub genes (CLEC5A, KLRB1, LCN2, MCEMP1 and MMP9) as potential candidate biomarkers (Supplementary Table S3).

### 3.6 Diagnosis value evaluation

**FIGURE 6:**
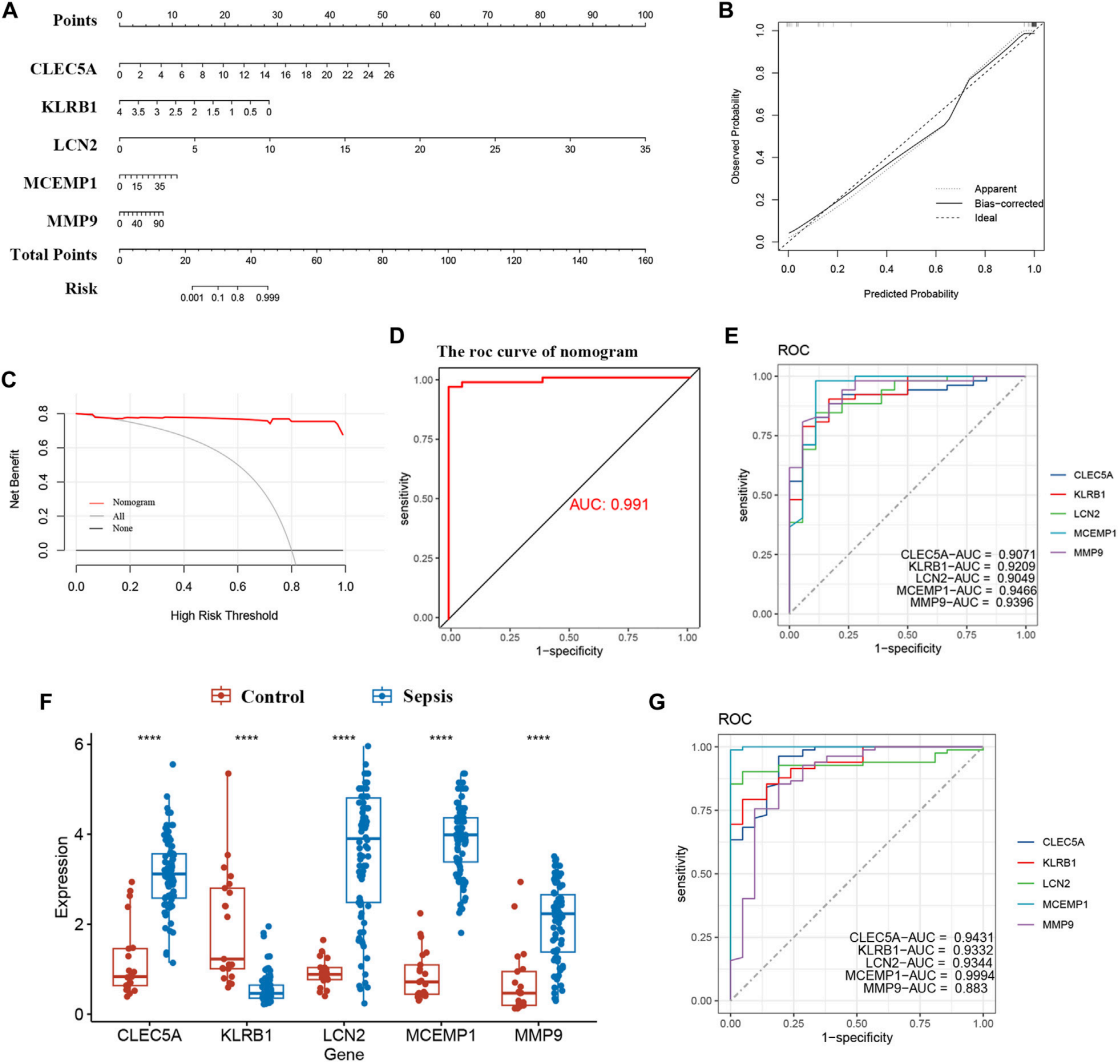
Nomogram construction and the diagnostic value evaluation. **(A)** Nomogram for the diagnostic model. **(B)** Calibration curve. **(C)** DCA for the diagnostic model. **(D, E)** The ROC curve of nomogram and each candidate gene (CLEC5A, KLRB1, LCN2, MCEMP1, and MMP9). **(F)** Validation of candidate hub genes in the GSE26378 dataset. **(G)** ROC curve of candidate hub genes in the GSE26378 dataset. *, *p* < 0.05, **, *p* < 0.01, ***, *p* < 0.001, ****, *p* < 0.0001.

To better diagnose and predict the five hub genes, we constructed a nomogram (Figure 6A). The calibration curve demonstrated that the predicted probabilities of our nomogram diagnostic model were in close agreement with the ideal model’s predicted probabilities (Figure 6B). Additionally, decision curve analysis (DCA) was performed on the nomogram, indicating that using the nomogram model for decision-making may be beneficial for diagnosing ROP with sepsis (Figure 6C). We calculated the AUC values for both the nomogram and each hub gene as follows: nomogram (AUC: 0.991), CLEC5A (AUC: 0.9071), KLRB1 (AUC: 0.9209), LCN2 (AUC: 0.9049), MCEMP1 (AUC: 0.9466), and MMP9 (AUC: 0.9396) (Figures 6D, E). As observed, all five candidate biomarkers had AUC values greater than 0.9, and the AUC value of the nomogram was higher than that of each individual hub gene, suggesting that the nomogram has strong diagnostic value for ROP with sepsis.

Furthermore, we validated five candidate biomarkers using the GSE26378 dataset. CLEC5A, LCN2, MCEMP1, and MMP9 exhibited significantly higher expression levels in patients, while KLRB1 showed markedly lower expression in patients (Figure 6F). Subsequently, we conducted ROC curve single-factor analysis on the GSE26378 dataset, resulting in AUC values of 0.9431 for CLEC5A, 0.9332 for KLRB1, 0.9344 for LCN2, 0.9994 for MCEMP1, and 0.883 for MMP9 (Figure 6G). These findings indicate substantial diagnostic potential for these five candidate biomarkers.

### 3.7 Immune infiltration analysis

**FIGURE 7:**
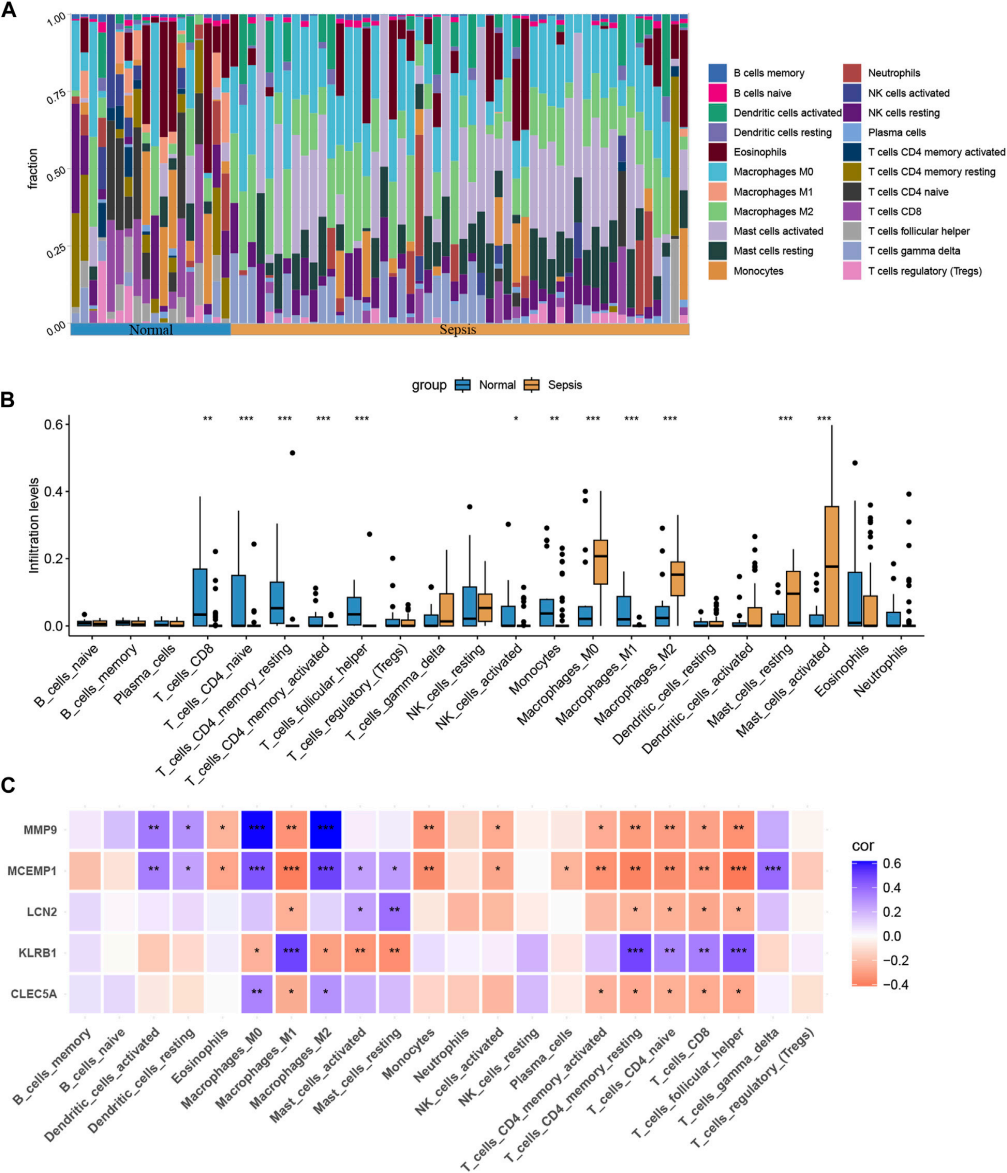
Immune cell infiltration analysis between sepsis and normal. **(A)** The proportion of 22 immunocytes in different samples visualized from the bar plot. **(B)** Comparison regarding the proportion of 22 kinds of immunocytes between sepsis and normal groups. **(C)** The correlation plot illustrates the association between different immune cell infiltrates and the five hub genes. *, *p* < 0.05, **, *p* < 0.01, ***, *p* < 0.001.

Our findings suggest that genes associated with ROP can also play a role in sepsis, particularly in immune regulation. Therefore, we proceeded with immune cell infiltration analysis to gain further insights into the involvement of the immune system in sepsis. Figure 7A displays the proportions of 22 immune cell types in samples from the sepsis group and the normal group. The box plots demonstrate that sepsis patients have higher levels of Macrophages M0, Macrophages M2, Mast cells resting, and Mast cells activated, while lower levels of T-cells CD8^+^, T-cells CD4 naive, CD4 memory resting, and T-cells follicular helper (Figure 7B). Furthermore, we observed significant correlations between the five hub genes and immune cell infiltration in sepsis, as depicted in Figure 7C. In conclusion, various immune cell types exhibit varying degrees of infiltration in sepsis patients, which may provide potential targets for novel therapies.

### 3.8 Identifying core small molecule compounds for treatment

**FIGURE 8:**
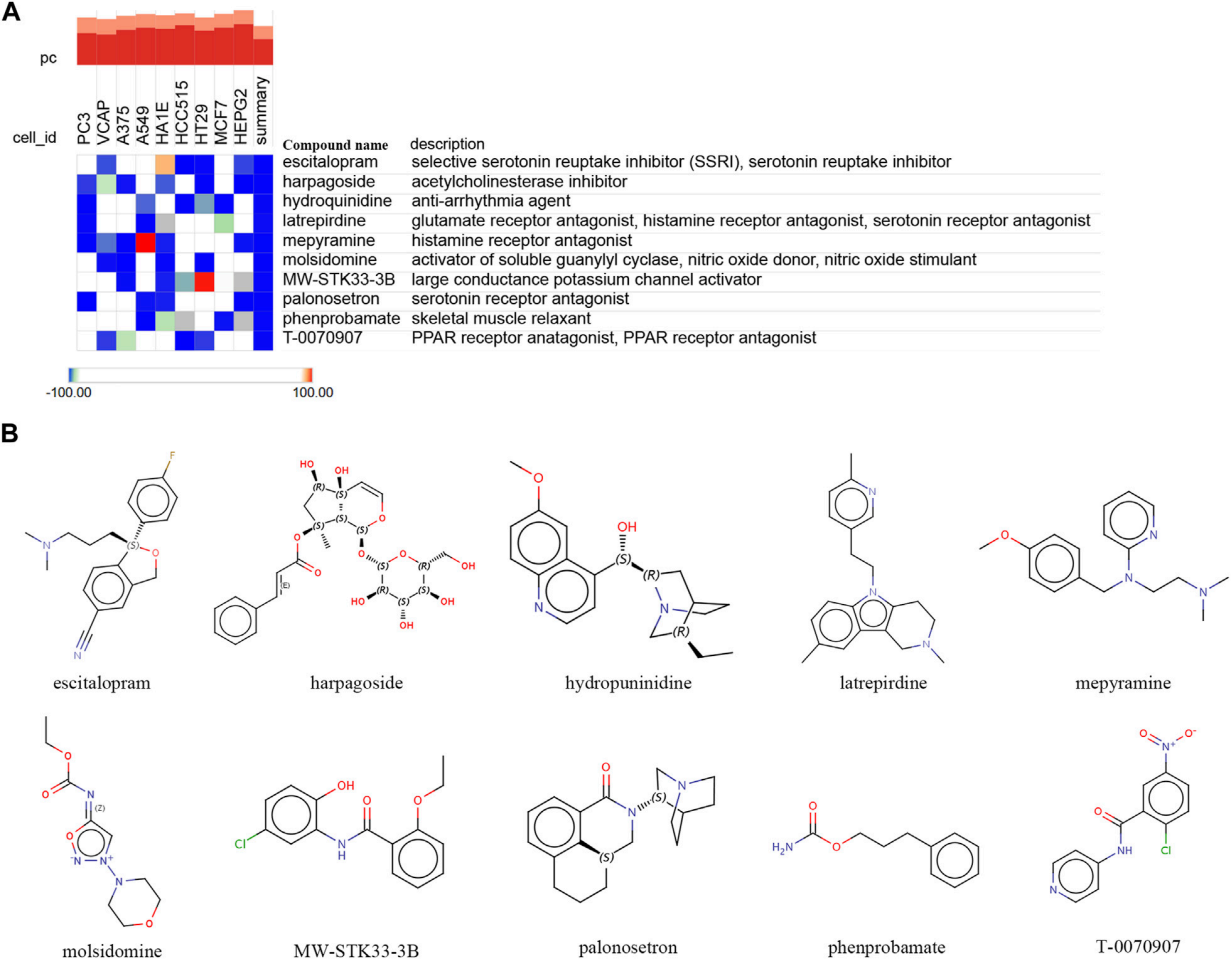
Identifying potential small-molecule compounds for the treatment of sepsis via cMAP analysis. **(A)** A heatmap illustrates the top 10 negatively enriched compounds. **(B)** The chemical structures of these 10 compounds.

To further explore potential small-molecule drugs that may have therapeutic effects for ROP with sepsis, we inputted the upregulated genes from the 34 overlapping genes into the cMAP database. Our aim was to identify small-molecule compounds that could potentially reverse the abnormal gene expression patterns. The top-scoring compounds with the most antagonistic effects included escitalopram, harpagoside, hydroquinidine, latrepirdine, mepyramine, molsidomine, MW-STK33-3B, palonosetron, phenprobamate, and T-0070907. These compounds displaying antagonistic effects may serve as potential therapeutic drugs (Figure 8A). Figure 8B displayed chemical structures of these ten small molecule compounds.

## 4 Discussion

ROP is a retinal vascular regulatory disorder that affects the development of the retinal vasculature in premature infants, and it is influenced by various factors, including infection and prematurity (Huang et al., 2019; Wang et al., 2019). Sepsis increases the risk and severity of ROP at any stage, and the inflammatory factors triggered by sepsis may regulate retinal vascular development and alter angiogenesis (Dong et al., 2020). Alterations in retinal vascular development during the developmental process contribute to the development of ROP, making it a prominent cause of visual impairment in children (Hong et al., 2014). Currently, researches combining these two diseases is relatively rare. Accordingly, we conducted bioinformatics analysis and machine learning methods to develop a nomogram for evaluating the diagnostic performance of ROP with sepsis. Interestingly, we discovered five key immune-related candidate hub genes (CLEC5A, KLRB1, LCN2, MCEMP1, and MMP9) and built a nomogram for this purpose.

C-Type lectin receptor 5A (CLEC5A), also known as MDL-1, promotes extracellular trap formation, reactive oxygen species generation, and the production of pro-inflammatory cytokines in neutrophils (Bakker et al., 1999; Chen et al., 2017). CLEC5A is primarily expressed on myeloid cells (neutrophils, monocytes, macrophages, and dendritic cells) and its expression is regulated by the key transcription factor PU.1, indicating that CLEC5A expression is modulated by oxidative stress (Cheung et al., 2011; González-Domínguez érika et al., 2015; Cheng et al., 2016). Furthermore, CLEC5A can be considered as an innate immune checkpoint that amplifies pro-inflammatory signals to facilitate the occurrence of infection or sterile inflammation (Tosiek et al., 2022). In sepsis, uncontrolled immune responses lead to harmful and potentially fatal inflammatory cascades. The involvement of CLEC5A in extracellular trap formation may exacerbate tissue damage and impede proper blood flow, potentially worsening the condition. In premature infants, the role of CLEC5A in modulating inflammatory responses in myeloid cells may contribute to abnormal retinal blood vessel development and neovascularization, leading to ROP.

Killer cell lectin-like receptor B1 (KLRB1), encodes a C-type lectin receptor found on natural killer cells and T-cells in peripheral blood, umbilical cord blood, and thymus (Giorda et al., 1990; Lanier et al., 1994; Duurland et al., 2022). According to research findings, KLRB1 plays a crucial role in differentiation, particularly in dendritic cells and monocytes, and the expression of KLRB1 can serve as an indicator of NK cells involved in the pathogenesis of inflammatory diseases (Poggi et al., 1997; Kurioka et al., 2018). The increased expression of KLRB1 on NK cells and T cells may be correlated with immune cell activation and inflammatory responses in sepsis. NK cells release cytokines and participate in cytotoxicity during inflammation, potentially influencing vascular development and inflammatory responses.

Lipocalin-2 (LCN2) is an innate immune protein involved in various physiological and pathological processes, including iron homeostasis, inflammation, and tumorigenesis (Chakraborty et al., 2012; Moschen et al., 2017). Additionally, we have found a clinical correlation between LCN2 and sepsis of intestinal origin (Lu et al., 2019). LCN2, as a transporter of lipids and iron, plays a critical regulatory role in lipid metabolism (Jaberi et al., 2021). Recent research has revealed its involvement in lipid metabolism dysregulation in various sepsis conditions (Amunugama et al., 2021). Moreover, LCN2 may be implicated in septic cardiomyopathy by mediating lipid accumulation and influencing mitochondrial function (Liu et al., 2022).

MCEMP1 is a transmembrane protein spanning the cell membrane and is commonly expressed by immune-related cells like mast cells and macrophages, playing a role in the pathogenesis of allergic and inflammatory diseases (Li et al., 2005; Schleinitz, 2015). Previous research has revealed that MCEMP1 plays a crucial role in sepsis and viral infections (Raman et al., 2016; Nicolas De Lamballerie et al., 2021). Therefore, we also consider it as a diagnostic biomarker.

MMPs are a zinc enzyme family that plays a critical role in degrading and remodeling extracellular matrix proteins during normal developmental processes, as well as being involved in various physiological functions such as inflammatory responses, organ morphogenesis, and vascular formation during pathological processes (Stamenkovic, 2003). MMP9 is predominantly released by neutrophils and macrophages, and it is responsible for regulating inflammation in various tissues and diseases (Deryugina et al., 2014). Interestingly, it has been observed that MMP9 is closely associated with the human protein homolog of LCN2, known as NGAL (Kjeldsen et al., 1993). The MMP9/NGAL complex exhibits a strong correlation with TSP1, and they are found to be more actively involved in the process of angiogenesis during orthodontic periodontal remodeling (Surlin et al., 2014). This finding suggests a potential interplay between MMP9/NGAL and TSP1 in the regulation of vascular formation. Furthermore, several studies have indicated that the expression and activity of MMPs increase during preterm birth, and they play a crucial role in fetal development, inflammatory responses, and angiogenesis (Cockle et al., 2007; Qazi et al., 2009). The imbalance between MMPs and their tissue inhibitors may also contribute to the occurrence of complications in newborns (Cockle et al., 2007). In the OIR model, we observed that systemic inhibition of metalloproteinases reduces neovascularization (Das et al., 1999). We speculate that MMP9 may be a candidate diagnostic gene for ROP patients with sepsis.

From previous research, we have come to understand that immune cells and inflammatory responses play a crucial role in the development and progression of sepsis (Riedemann et al., 2003). The core mechanism of sepsis is immune dysfunction, and as key cells in the innate immune system, macrophages play a crucial role in sepsis by performing functions such as antigen presentation and secretion of inflammatory and chemotactic factors (Epelman et al., 2014; Luan et al., 2014; Qiu et al., 2019). Autophagy is closely linked to inflammation and immunity, and enhancing autophagy in sepsis can exert a protective effect by negatively regulating aberrant macrophage activation, reducing inflammasome activation and the release of pro-inflammatory cytokines, and influencing macrophage apoptosis (Qiu et al., 2019). The majority of studies emphasize the role of mast cells in the early stages of sepsis, where they exhibit immediate inflammatory responses and unique potential for combating infections (Gekara and Weiss, 2008; Heib et al., 2008; Ramos et al., 2010). Furthermore, we observed that inducing sepsis in mice leads to a decrease in the percentage of CD4^+^ T-cells, while the percentages of T helper cells (Th2 and Th17) and regulatory T-cells (Treg) are upregulated (Yeh et al., 2022). In our study, we observed elevated levels of macrophages and mast cells, while the levels of CD8^+^ T-cells, naive CD4^+^ T-cells, memory CD4^+^ T-cells, and follicular helper T-cells were found to be lower, which is consistent with previous research findings. Furthermore, a recent study suggested that immune cells might have played a role in the onset and progression of pediatric sepsis, which aligns with our findings (Zhang et al., 2023). By gaining a deeper understanding of the regulation mechanisms of immune cells and inflammatory responses, we can better comprehend the pathophysiology of sepsis, thus providing more comprehensive and precise guidance for the search of effective therapeutic strategies.

In recent years, significant progress has been made in identifying small molecule compounds with therapeutic potential for various diseases. Compared to other treatment methods, small molecule compounds offer advantages such as strong penetrability, high specificity, convenient administration, tunable pharmacokinetic properties, diversity, and flexibility, making them promising candidates for disease treatment. A metal gel capable of inhibiting sepsis was successfully synthesized through direct coordination interactions and Schiff base reactions between aminoglycosides, 2,2′-bipyridine-4,4′-dicarbaldehyde, and metal ions (Li et al., 2023). However, to date, there has been no research focusing on identifying potential therapeutic small molecule compounds based on sepsis gene expression characteristics. Therefore, we conducted a cMAP analysis, linking the upregulated genes in sepsis with the pathogenic genes related to ROP, and identified ten small molecule compounds (escitalopram, harpagoside, hydroquinidine, latrepirdine, mepyramine, molsidomine, MW-STK33-3B, palonosetron, phenprobamate, and T-0070907) as candidate compounds for potential sepsis treatment. In the cMAP analysis, these small molecule compounds exhibited the highest negative enrichment score, indicating that it has the potential to effectively reverse the upregulated expression of pathogenic genes in sepsis.

## 5 Conclusion

By leveraging bioinformatics analysis and machine learning algorithms, we systematically identified five immune-related candidate hub genes (CLEC5A, KLRB1, LCN2, MCEMP1, and MMP9) and provided a nomogram for diagnosing ROP with sepsis. Additionally, we emphasized the imbalance of immune cells and uncovered small molecule compounds as highly promising candidates for sepsis treatment based on our analysis with cMAP. In conclusion, our research offered comprehensive insights into the pathophysiology and treatment of ROP with sepsis, ranging from the discovery of immune-related candidate genes, the construction of predictive models, to the identification of immune cell dysregulation and potential therapeutic drugs.

## Data Availability

The original contributions presented in the study are included in the article/Supplementary Material, further inquiries can be directed to the corresponding author.
